# Annotations

**Published:** 1904-08-20

**Authors:** 


### Annotations.

### The New Regius Professor of Physic at Oxford.

The announcement that Dr. William Osler, F.R.S., has been appointed Regius Professor of Physic at Oxford will be heartily welcomed both in the University and by the medical profession. Professor Osler succeeds Sir John Burdon Sanderson, who himself followed Sir Henry Acland in a post of great dignity but small emolument. Professor Osler is by birth a Canadian, and graduated in the McGill University in 1872. He afterwards taught physiology and pathology at his alma mater. Twelve years later he migrated to the University of Pennsylvania, where he occupied the chair of clinical medicine, and since 1889 he has been Professor of the Principles and Practice of Medicine at the Johns Hopkins University and physician to the Johns Hopkins Hospital, Baltimore. Professor Osler is as well and favourably known in England as he is at home; in coming to Oxford, therefore, *mutat coelum non amicos*, and we bid him a most hearty welcome and a very long tenure of office. A few weeks ago, on the occasion of the visit of the British Medical Association to Oxford, he received the degree of D.Sc., *honoris causâ*, and was described by Professor Love as having been for many years a leading exponent of the principle that the art of medicine should be based upon the most exact scientific knowledge of the day. Professor Osler married, in 1892, Grace, eldest daughter of the late Mr. John Revere, of Boston, and widow of Dr. G. W. Gross, of Philadelphia. For the University his acceptance of the position of official head of the medical school is an event of the highest importance. The laboratories and the intellectual material are present in abundance, the controlling force has alone been wanting. Professor Osler comes fresh from one of the greatest and most modern schools of medical research, and he brings with him methods as yet untried in England. He is of an age which allows him to take an active part in the work of teaching, directing, and organising, whilst at the same time he is endowed with the tact which is above all things necessary to bring the work of his subordinates to an harmonious issue. In Oxford there is a real danger of becoming so immersed in University politics that the real objects in life are lost sight of. If Professor Osler is able to avoid this pitfall he should be able to place the Oxford Medical School in the position it has not occupied since the foundation of the Royal Society, a position which was then characterised by breadth of thought and originality of research.

### The Inspection of Schools.

The washing of dirty linen before the public gaze is hardly an æsthetic or a pleasing process, but it sometimes leads to discoveries which are an advantage to the public interest. At the present moment the community generally is for various reasons much concerned with the efficiency of elementary education, and some recent utterances regarding the sanitary condition of many of the buildings in which this education is conducted are calculated to cause considerable uneasiness. It has been a comfortable conviction of the patriotic citizen that both the educational discipline and the sanitary arrangements of the elementary schools were under the keen eyes of a body of inspectors whose independent reports were the free utterances of impartial experts. But if a letter which has appeared in the public prints signed by the late Secretary of the Board of Education is to be accepted as gospel, this pleasing creed must be abandoned as a delusion. The inspectors, we are told, find themselves compelled to make favourable reports on school buildings, or at all events to abstain from the opposite course, because anything like harsh criticism is apt to bring a deputation "politically important and denominationally influential" to the Education Department, and the critic discovers that his criticisms are ignored. But, according to the indictment framed by the Secretary, not only are the reports made by the inspectors unreliable in this respect, but many of the school buildings are absolutely insanitary and prejudicial to the health of the children attending them. This applies, it is said, to "hundreds of schools throughout the country" which have been spared "on account of the managers' pockets and denominational instruction." We have nothing here to do with the suggested motive for such proceedings, but as a matter concerning the health of large numbers of the rising generation, we are free to say that Sir George Kekewich's letter announces a scandal of the first order. If his charges can be substantiated, the public confidence has been abused in the most shameful fashion and public money has been systematically misapplied. It is impossible the matter can rest in its present position. Charges of such gravity endorsed by so weighty a name must be probed to the bottom.

### Mistaken Identity.

The numerous cases of mistaken identity which have recently been recorded in the Press have not only aroused curiosity, but have produced in some minds a sense of anxiety or even of insecurity. If a perfectly innocent man can on two separate occasions be convicted of grave crime on evidence which for all practical purposes was evidence of identification, it can hardly be denied that there is some room for concern. This position has been emphasised by various accounts of other mistakes and mishaps due to the existence of "doubles," and by one or two examples of people committing gross blunders in the identification of dead bodies. All this to students of medical jurisprudence is nothing new, and the essentially small proportion of blunders made in the total of identification processes is full of practical comfort. At the same time the error, when it occurs even in a single case, may have such disastrous consequences for the individual that every possible care ought to be taken to guard against it. More particularly it may be urged that the police methods of securing identification of a suspected offender by various witnesses should be brought under review. Further, it is fair to remember that identification which depends on what are called "general appearances," is of much less value than when some specific distinctive point can be quoted in support of the opinion. After all, it is not to be wondered at that cases of mistaken identity occur, for the majority of witnesses are not persons who have cultivated the habit of accurate observation, and the "method of Zadig" is not specially honoured or practised amongst us.

